# Development of a baculovirus vector carrying a small hairpin RNA for suppression of sf-caspase-1 expression and improvement of recombinant protein production

**DOI:** 10.1186/s12896-018-0434-1

**Published:** 2018-05-02

**Authors:** Xiaoyue Zhang, Keyan Xu, Yanmei Ou, Xiaodong Xu, Hongying Chen

**Affiliations:** 1 College of Life Sciences, Northwest A & F University, Yangling, Shaanxi 712100 People’s Republic of China; 2 Innovative Experimental College, Northwest A & F University, Yangling, Shaanxi 712100 People’s Republic of China

**Keywords:** Baculovirus expression vector system, Bacmid, Sf-caspase-1, shRNA, RNA interference

## Abstract

**Background:**

The Baculovirus expression vector system (BEVS) is a transient expression platform for recombinant protein production in insect cells. Baculovirus infection of insect cells will shutoff host translation and induce apoptosis and lead to the termination of protein expression. Previous reports have demonstrated the enhancement of protein yield in BEVS using stable insect cell lines expressing interference RNA to suppress the expression of caspase-1.

**Results:**

In this study, short-hairpin RNA (shRNA) expression cassettes targeting *Spodoptera frugiperda* caspase-1 (Sf-caspase-1) were constructed and inserted into an *Autographa californica* multiple nucleopolyhedrovirus (AcMNPV) vector. Using the recombinant baculovirus vectors, we detected the suppression of Sf-caspase-1 expression and cell apoptosis. Green fluorescent protein (GFP), *Discosoma sp.* Red (DsRed) and firefly luciferase were then expressed as reporter proteins. The results showed that suppression of apoptosis enhanced the accumulation of exogenous proteins at 2 and 3 days post infection. After 4 days post infection, the activity of the reporter proteins remained higher in BEVS using the baculovirus carrying shRNA in comparison with the control without shRNA, but the accumulated protein levels showed no obvious difference between them, suggesting that apoptosis suppression resulted in improved protein folding rather than translation efficiency at the very late stage of baculovirus infection.

**Conclusions:**

The baculovirus vector developed in this study would be a useful tool for the production of active proteins suitable for structural and functional studies or pharmaceutical applications in *Sf*9 cells, and it also has the potential to be adapted for the improvement of protein expression in different insect cell lines that can be infected by AcMNPV.

## Background

Baculoviruses have been widely used as vectors for protein expression since the generation of a recombinant *Autographa californica* multiple nucleopolyhedrovirus (AcMNPV) for human IFN-β expression was reported in 1983 [1]. The insect cell based baculovirus expression vector system (BEVS) has been developed for a number of baculovirus species including AcMNPV, *Bombyx mori* nucleopolyhedrovirus (BmNPV) [2] and *Spodoptera exigua* multiple nucleopolyhedrovirus (SeMNPV) [3]. BEVS possesses advantages such as abundant protein expression level, proper post-translational modification, and lower cost than mammalian cell expression. There are two commonly used strategies, by direct insertion of foreign genes into a baculovirus shuttle vector (bacmid) in *Escherichia coli* [4] or co-transfection of insect cells with linearized baculovirus DNA (or replication-deficient bacmid) and a transfer plasmid carrying the gene of interest [5–7], for the construction of baculovirus vectors.

To improve the quantity and quality of recombinant proteins produced in BEVS, considerable efforts have been made to modify baculovirus vectors in the past 30 years [8, 9]. In the baculovirus replication cycle, the viruses can produce two forms of virions, the occlusion derived virus (ODV) and the budded virus (BV). The ODV is responsible for primary infection in host midgut tissue and commonly used as an insecticide [10], while the BV infects all other host tissues in natural infections and is used for the infection of cultured cells in BEVS. A number of ODV-associated proteins are dispensable for BV production and overexpression of recombinant proteins in cultured cells. Baculoviruses used for recombinant protein production usually lack the polyhedrin gene (*polh*) encoding for the occlusion body (OB) matrix protein, using the *polh* locus for foreign gene insertion. In addition to *polh*, the virus chitinase (*chiA*) and cathepsin (*v-cath*) genes involved in the release of OB are also dispensable for the virus replication in cultured insect cells. It has been reported that removal of baculovirus *chiA* and *v-cath* can improve the stability of exogenous proteins in different insect cells including *S. frugiperda* (Sf), *B. mori* (Bm), and *T. ni* (Tn) High Five™ cells [11–13]. AcMNPV *p10*, which is abundantly expressed very late during infection and may compete with *polh* at a transcriptional level, together with its flanking genes p26 and p74, can also be deleted for enhancement of recombinant protein expression [8].

Modification of foreign gene expression cassettes is another approach to increase recombinant protein production. The strong baculovirus late gene promoters of *polh* and *p10* are the most commonly used controlling elements for efficient expression of foreign genes in BEVS. Earlier promoters have been utilized for heterologous protein production, but result in reduced productivity [14]. In a recent report, a novel expression cassette was designed to overexpress baculovirus transactivation factors IE1 and IE0, and contained an enhancer sequence (hr1) and promoters interacting with each other and acting in a cascade. The recombinant protein production dramatically increased using this vector as compared to a conventional baculovirus vector [15]. Other reports have shown that fusion of foreign proteins to tags such as maltose binding protein [16], SUMO [17] and KDEL retention signal [18] can also improve protein production in BEVS.

BEVS is a transient expression system, the death of baculovirus-infected insect cells terminates protein production which peaks around 3 days post infection. Methods to slow cell death would enhance the productivity of this technology. It has been reported that introducing of vankyrin from the insect virus *Campoletis sonorensis* ichnovirus can delay lysis of baculovirus-infected cells and result in an increase of protein yield [19].

Apoptosis, characterized by a series of morphological and biochemical features that are associated with cell death, is an important host defense mechanism to limit and control viral infections. Baculovirus replication in insect cells triggers shutoff of host protein synthesis and induces apoptosis [20], which in turn reduces both virus replication in cultured cells and virus infectivity and transmission in the host [21–23]. To counteract this cellular defense reaction, baculoviruses have evolved to encode anti-apoptotic genes including *p35*, *p49*, *iap* (inhibitor of apoptosis), and *apsup* (apoptosis suppressor) [23–26]. These anti-apoptotic genes play an important role in baculovirus infections, and it has been reported that the infectivity of AcMNPV mutants lacking *p35* can reduce more than 1000-fold [21, 27].

The Apoptotic process involves the activation of a series of proteases called caspases. Blocking the activity of caspases by RNA interference have been used to improve protein production in mammalian cells [28, 29]. In BEVS, several reports have established stable insect cell lines expressing short-hairpin RNAs (shRNA) [30] or long double stranded RNA (dsRNA) that can be processed by Dicer into siRNAs of 21-23 bp [31–33] to suppress the expression of caspase-1 and detected the improvement of protein production.

In this study, we constructed shRNA expression cassettes targeting *Spodoptera frugiperda* caspase-1 (Sf-caspase-1) and engineered the cassettes into a baculovirus vector. Using the recombinant baculovirus vectors, we detected the suppression of Sf-caspase-1 expression and cell apoptosis, and examined the production of reporter proteins green fluorescent protein (GFP), *Discosoma* sp. red fluorescent protein (DsRed) and firefly luciferase, in comparison with BEVS without shRNA.

## Methods

### Viruses and cell lines

The bacmid BAC10:KO1629, which contains an AcMNPV genome, was maintained in *E*. *coli* strain HS996 cells as previously described [6]. *Spodoptera frugiperda* 9 (*Sf*9) cells (Invitrogen) were cultured in T25 Flasks or 6-well plates at 27 °C in SFX insect medium (HyClone, GE healthcare life science) containing 1% fetal bovine serum (HyClone).

### Plasmid construction

To make the constructs expressing short hairpin RNAs (shRNA), human U6 promoter was amplified by PCR from the genomic DNA of human embryonic kidney 293 T cells (the Cell Bank of the Chinese Academy of Sciences), using primers HU6pF (5’-ACCACCGCGGAGGTCGGGCAGGAAGAGGGCCTATTT-3′, with underlined *Sac*II site) and HU6pR (5’-CTAGTCTAGAGCCATTGGCTGCAGTACTTCGTCCTTTCCACAAGATA-3′, with underlined *Xba*I and *Pst*I sites). The amplified DNA fragment was digested with double restriction enzymes *Sac*II and *Xba*I and inserted into pTriEx-GFP [34], and the resulting construct was named pTriEx-U6-GFP.

To suppress the expression of *Spodoptera frugiperda* caspase-1 (Sf-caspase-1), three pairs of oligomers were synthesized (sequences are listed in Table 1) to make the constructs expressing three shRNAs, which were designed based on a previous report and comprised of a 21 base pair stem (underlined in Table 1) and a 9 nucleotide loop region [35]. The forward and reverse oligomers for each shRNA were annealed to form cohesive ends. The double-stranded oligonucleotides were inserted into pTriEx-U6-GFP between the *Pst*I and *Xba*I sites, generating constructs pTriEx-U6Si1-GFP, pTriEx-U6Si2-GFP and pTriEx-U6Si3-GFP.

**Table 1:** Oligonucleotides used for inserting of shRNA genes

Name	Sequence
Si1F	5’-GGCTAGGATGCCAGTTGATAGATTAATAAGCTCTATCAACTGGCATCCTAGCAAAAAT-3’
Si1R	5’-CTAGATTTTTGCTAGGATGCCAGTTGATAGAGCTTATTAATCTATCAACTGGCATCCTAGCCTGCA-3’
Si2F	5′- GGCTGTATGCCAAAGATACTCATTAATAAGCTGAGTATCTTTGGCATACAGCAAAAAT-3’
Si2R	5′- CTAGATTTTTGCTGTATGCCAAAGATACTCAGCTTATTAATGAGTATCTTTGGCATACAGCCTGCA-3’
Si3F	5′- GGCTAGACTTTGAGTCTAATGCTTAATAAGCGCATTAGACTCAAAGTCTAGCAAAAAT-3’
Si3R	5’-CTAGATTTTTGCTAGACTTTGAGTCTAATGCGCTTATTAAGCATTAGACTCAAAGTCTAGCCTGCA-3’

To investigate the effects of Sf-caspase-1 suppression on protein expression in BEVS, the genes for GFP, DsRed and firefly luciferase were amplified using the primers described before [36, 37] and cloned into the expression vector pTriEx1.1.

### Modification of bacmid and generation of recombinant baculoviruses

**Fig. 1:**
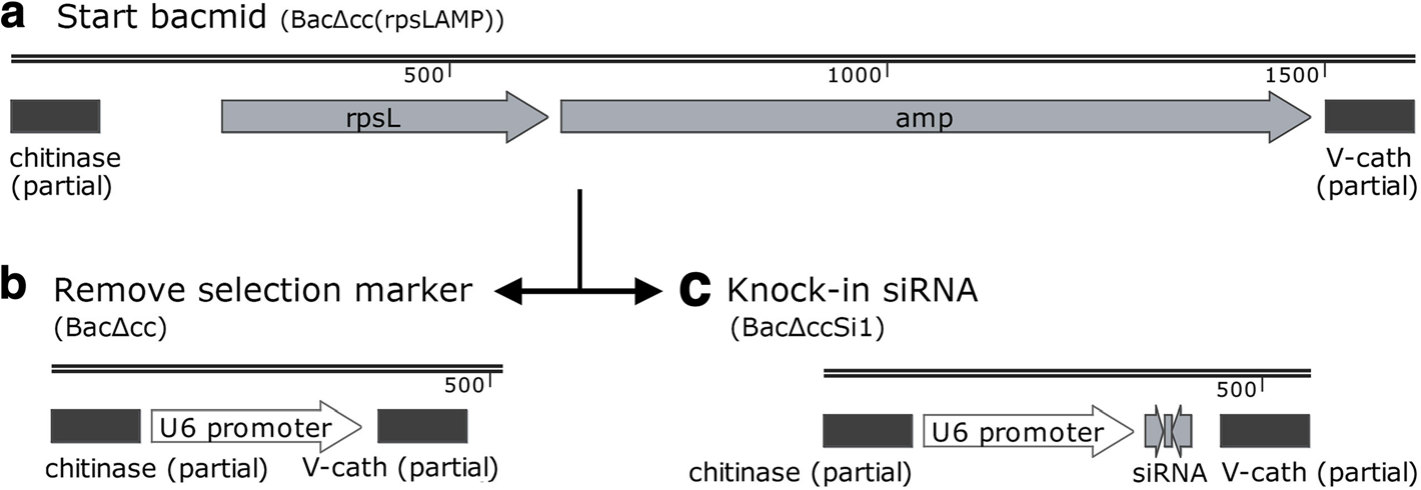
Strategy for construction of baculovirus vectors BacΔCC(rspLamp) (**a**), BacΔCC (**b**) and BacΔCCSi1 (**c**)

The *chitinase* and *v-cathepsin* genes (*ChiA/V-Cath*) were knocked out from the bacmid BAC10:KO1629 by Red/ET-based recombination. Briefly, rpsL-amp cassette was amplified by PCR using primers ChiF_rpsL (5’-CGAGTTCCAACTTGGCGCGTAGACTGTTGTTTGGTAGCCCAAATCCGTGAGATGGCCTGGTGATGG-3′) and ChiR_AMP(5’-TGTTTATAGTTAACAATGTCGGCAGCGTCTATGGCCATAGGAATAGGGCCTTACCAATGCTTAATC-3′), which were comprised of a homologous arm for *ChiA/V-Cath* (underlined) and the rpsL-amp cassette. The amplified fragment was transformed into HS996 competent cells containing the Red®/ET® plasmid pSC101-BAD-gbaA and bacmid BAC10:KO1629, and the 1928 bp *ChiA/V-Cath* fragment was replaced by the rpsL-amp cassette [11]. The resulting bacmid BacΔCC(rspLamp) (Fig. 1a) was used as the start bacmid to knock in shRNA and make recombinant baculoviruses used in this study.

To knock in shRNA, the U6Si1 fragment containing the U6 promoter and shRNA1 gene and ChiA/V-Cath flanking sequences was amplified from the template pTriEx-U6Si1-GFP by PCR, using primers ChiF (5’-CGAGTTCCAACTTGGCGCGTAGACTGTTGTTTGGTAGCCCAAATCCGTGAAGGTCGGGCAGGAAGAGG-3′) and ChiR (5’-TGTTTATAGTTAACAATGTCGGCAGCGTCTATGGCCATAGGAATAGGGCCGGCATGAACATGGTTAGCAG-3′). The amplified fragment was transformed into HS996 competent cells containing the Red®/ET® plasmid pSC101-BAD-gbaA and bacmid BacΔCC(rspLamp), so that the rpsL-amp cassette could then be removed and replaced by U6Si1, and this generated BacΔCCSi1 with the knock-in shRNA1 gene in the bacmid backbone (Fig. 1c). The fragment amplified from template pTriEx-U6-GFP using primers ChiF and ChiR was also used to replace the rpsL-amp cassette, resulting the control bacmid BacΔCC (Fig. 1b).

Recombinant baculoviruses were produced by homologous recombination between co-transfected pTriEx-1.1 (Novagen) based plasmid and linearized bacmid using FuGENE HD Transfection Reagent (Promega). Baculoviruses generated by homologous recombination were harvested at 96 h post transfection (hpi), passaged two times, and titrated by a 50% tissue culture infective dose (TCID_50_) endpoint dilution assay.

### Semi-quantitative RT-PCR

To detect the transcription level of Sf-caspase-1, total RNAs were isolated with the TRIzol RNA extraction kit (Beijing CoWin Biotech) from *Sf*9 cells infected by recombinant viruses at 48 hpi. The RNA samples were then treated with RNase-Free DNaseI (Thermo), and 2 μg of total RNA was used for reverse transcription performed using the 5× All-In-One RT MasterMix (ABM) according to the manufacturer’s instruction. The Sf-caspase-1 gene fragments were amplified by PCR using the primers Sf-casp1F (5’-TGTCAAACACCTTTTATG-3′) and Sf-casp1R (5’-TATTATGACACATGGGCA-3′). β-actin gene was also amplified as a control using primers actin-F(5′-GGTGGTTGTTAGATGTA-3′)and actin-R (5’-GCTGGGAGTGTATTTC-3′). After 25–29 cycles of amplification, the PCR samples were analyzed by 1% agarose gel electrophoresis.

### Analysis of apoptosis by Propidium iodide staining and flow cytometry

*Sf*9 cells were infected with recombinant baculoviruses at multiplicities of infection (MOI) of 5, harvested at 96 hpi by centrifugation at 500 g for 1 min, and then resuspended in PBS. Propidium iodide (PI) was added to the cell suspension to a final concentration of 10 μg/ml. After incubation at room temperature for 5 min in the dark, cells were analyzed by flow cytometry using a CyFlow Cube 6 flow cytometer (Partec).

### Protein expression and detection

*Sf*9 cells were infected by recombinant baculoviruses and incubated for 2–5 days at 27 °C, and then dislodged from the plates. The fluorescence of GFP and DsRed in the re-suspended cells was detected by flow cytometry [34], and the protein levels were determined by Western blot using anti-His monoclonal antibody (CoWin Biotech, China) [36]. Firefly luciferase protein level was analyzed by Western blot, and its enzyme activity was determined by Glomax 20/20 Luminometer (Promega), using a Luciferase Assay System (Promega). For the luciferase activity assay, samples were analyzed in triplicates, and each sample measurement was repeated three times.

## Results

### Silencing sf-caspase-1 via shRNA

Long dsRNAs have been shown to be able to be expressed and interfere with the transcription of caspase-1 in stably transfected Sf and Tn cells [31–33], and stable Bm cells expressing shRNAs have also been made to suppress the Bm-caspase-1 [30]. Suppression of the caspase-1 expression improved the production of recombinant proteins in these insect cells with BEVS. In order to investigate whether shRNAs could be directly delivered by a recombinant baculovirus and efficiently suppress Sf-caspase-1 expression, shRNAs with a 21 base pair stem and a 9 nucleotide loop region were designed and their coding sequences were cloned into a modified pTriEx vector under the control of the human U6 promoter. Since the U6 promoter sequence for *Sf*9 cell is still unknown and the human U6 promoter has been found to be able to drive shRNA expression in Tn insect cells as efficiently as *Drosophila* U6 promoter [38], human U6 promoter was used to control the shRNA expression in this study.

**Fig. 2:**
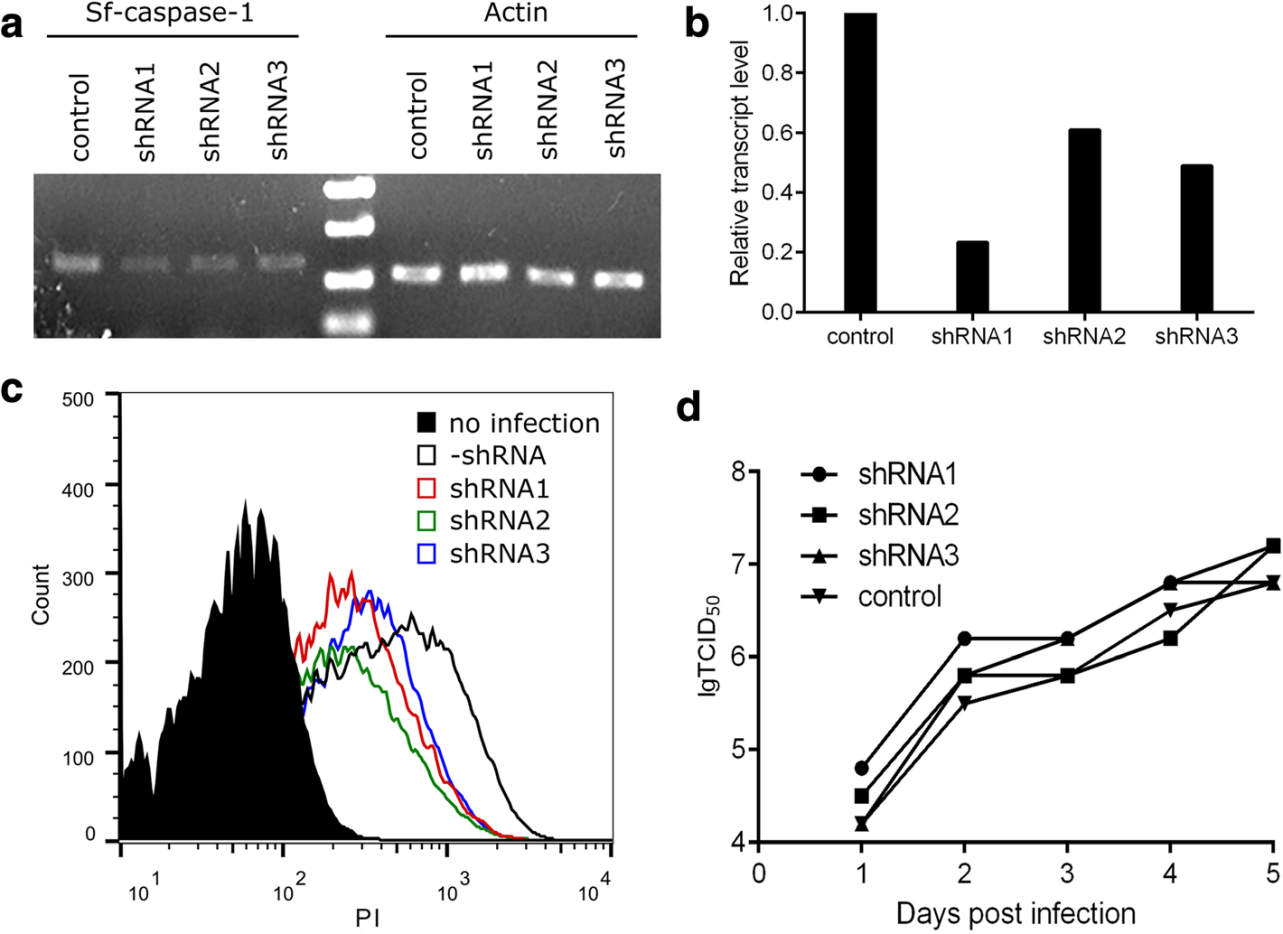
Suppression of Sf-caspase-1 by baculoviruses expressing shRNAs. **a** Determination of the transcript levels of Sf-caspase-1 by semi-quantitative RT-PCR. *Sf*9 cells infected with the baculoviruses expressing the indicated shRNA were analyzed at 2 dpi. The mRNA level of β-actin was detected as the control in each sample. **b** The relative transcript levels of Sf-caspase-1, which were normalized to the level of actin transcript in each sample. **c** Analysis of apoptosis by PI staining and flow cytometry. Infected cells were analyzed at 4 dpi. **d** BV titres of the recombinant baculoviruses

The plasmid constructs carrying the shRNA expression cassettes were co-transfected with AcMNPV bacmid into *Sf*9 cells to generate recombinant baculoviruses by homologous recombination. The transcript levels of Sf-caspase-1 in insect cells were determined by semi-quantitative RT-PCR at two days post-infection (dpi) of the recombinant baculoviruses. As shown in Fig. 2a and b, the transcript level of Sf-caspase-1 in the cells infected by the baculovirus expressing shRNA1, which targeted the 124–145 nucleotide site of the Sf-caspase-1 gene, decreased 76% compared to the control baculovirus infected cells. shRNA2 (targeting site 422–442) and shRNA3 (targeting site 783–803) also inhibited about 50% of the expression of Sf-caspase-1. Apoptotic analysis of the infected cells by PI staining and flow cytometry detected less late apoptotic cells in the groups infected by baculoviruses expressing shRNAs than the control group (Fig. 2c). Titration of the recombinant baculoviruses from 1 to 5 dpi revealed that shRNA delivery and expression did not affect the budded virus (BV) yield in *Sf*9 cells (Fig. 2d). These results demonstrated that shRNAs delivered by baculoviruses efficiently interfered with the transcription of Sf-caspase-1 and suppressed the cell apoptosis induced by the virus infection.

### Production of fluorescence proteins

It has been reported that deletion of *ChiA/V-Cath* could enhance protein production for both secreted and cytoplasmic proteins [11, 12]. To develop a baculovirus shuttle vector (bacmid) carrying the shRNA expression cassette, *ChiA/V-Cath* was knocked out from AcMNPV bacmid BAC10:KO1629 and used as the knock-in locus for the Sf-caspase-1 shRNA1, which inhibited Sf-caspase-1 expression best among the three shRNAs (Fig. 2a and b), generating the recombinant bacmid BacΔCCSi1. A control bacmid BacΔCC was made with an irrelevant knock-in fragment under the control of the human U6 promoter.

**Fig. 3:**
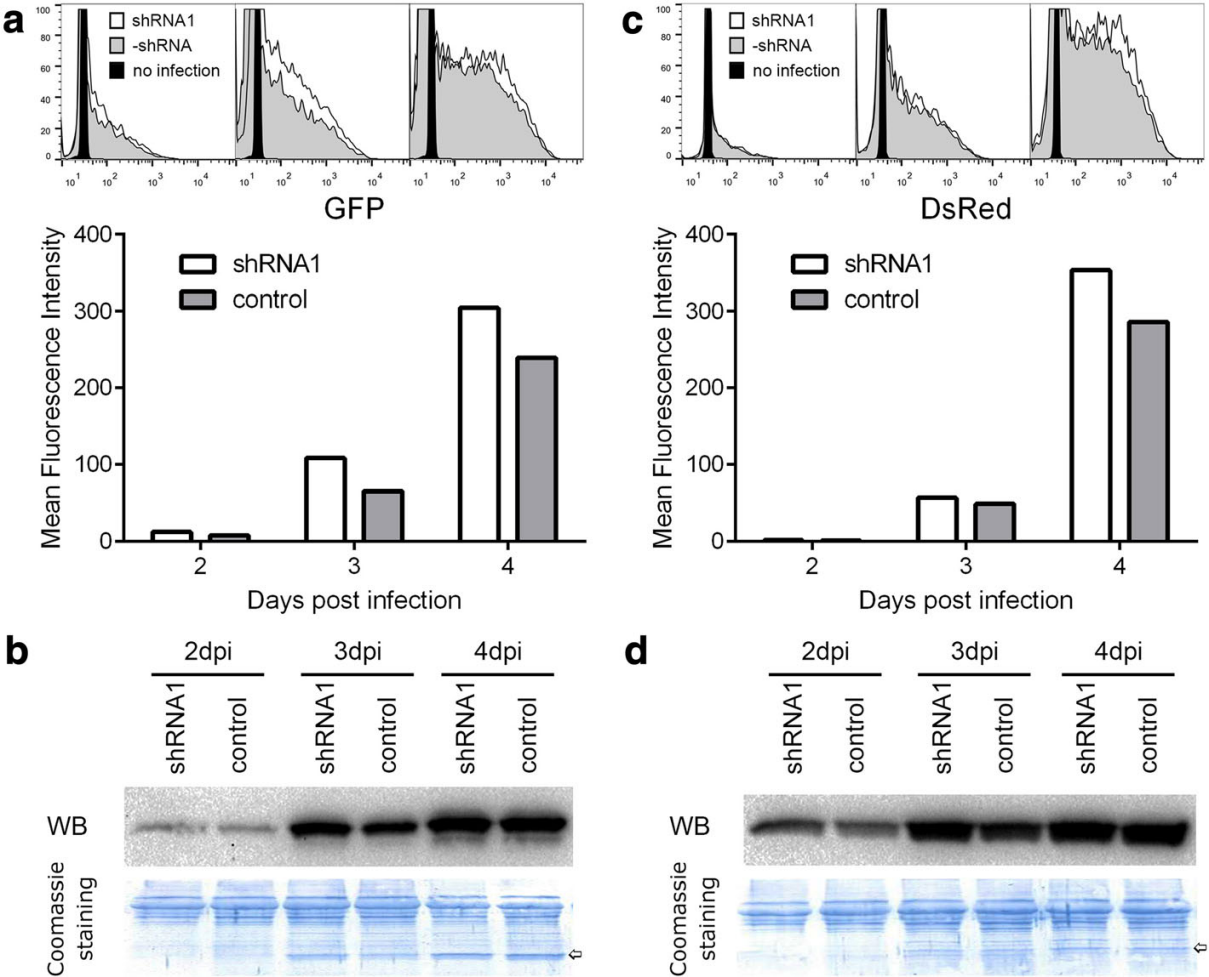
Production of fluorescence proteins using bacmid carrying the Sf-caspase-1 shRNA1 expression cassette. **a**. Detection of the GFP fluorescence by flow cytometry. Fluorescence histograms (upper panel) and the mean fluorescence intensities (lower panel) are shown. Data were collected from 3 × 10^4^ cells for each sample. **b**. Determination of the GFP protein levels by Western blot (upper panel). The recombinant GFP was probed by anti-His monoclonal antibody. The loaded total proteins on the PVDF membrane were visualized by coomassie brilliant blue R250 staining, and the GFP band on the stained membrane is indicated by an arrow (lower panel). **c**. Detection of the DsRed fluorescence by flow cytometry. **d**. Determination of the DsRed protein levels by Western blot (upper panel). The DsRed band on the stained PVDF membrane is indicated by an arrow (lower panel)

The bacmid BacΔCCSi1 carrying the Sf-caspase-1 shRNA1 expression cassette and control bacmid BacΔCC were respectively co-transfected with pTriEx-GFP into *Sf*9 cells to generate recombinant baculoviruses expressing GFP by homologous recombination. The GFP fluorescence of the baculovirus infected cells was measured by flow cytometry at 2, 3 and 4 dpi. The assay was repeated at least three times and the mean fluorescence intensity of the Sf-caspase-1 repressed cells were constantly shown to be stronger than the control baculovirus infected cells (typical data shown in Fig. 3a). However, the GFP protein levels in the apoptosis suppressed cells only slightly increased at 2 and 3 dpi when they were detected by Western blot, and no difference between apoptosis suppressed cells and the control was observed at 4 dpi (Fig. 3b).

It has been proved that exogenous protein production in BEVS could be improved in caspase-1 suppressed stable insect cells [30–33]. However, the protein levels were only reflected by fluorescence intensity or reporter enzyme activity in these studies. Here, we detected higher GFP fluorescence produced by an infection using a baculovirus expressing Sf-caspase-1 shRNA1 from 2 to 4 dpi, but it seemed that the accumulated GFP level was equivalent to the control by 4 dpi. It was reported that the enhanced production of exogenous proteins in BEVS was strongly associated with the retention of molecular chaperone expression in baculovirus-infected Sf-caspase-1 repressed stable cells [31]. Therefore, we speculated that the expression of chaperones in late baculovirus infections could benefit GFP folding and contribute to the stronger fluorescence intensity.

To further investigate the effect of Sf-caspase-1 suppression on fluorescent protein production, a red fluorescent protein DsRed, which is a tetramer and represents a protein where the maturation of the fluorophore is slow [39], was expressed using bacmid BacΔCCSi1 and BacΔCC. Like the GFP protein, the protein production of DsRed determined by Western blot was slightly higher in the apoptosis suppressed cells at 2 and 3 dpi, and no difference was detected by 4 dpi (Fig. 3d). By flow cytometry, the fluorescence intensity showed no obvious difference between the two groups at 2 and 3 dpi, but the signal was obviously stronger in shRNA expressing cells than the control by 4 dpi. These data suggested that suppression of apoptosis could enhance the accumulation of exogenous proteins in early infection and benefit the correct folding of proteins in the late stage of infection in BEVS.

### Expression of luciferase protein

**Fig. 4:**
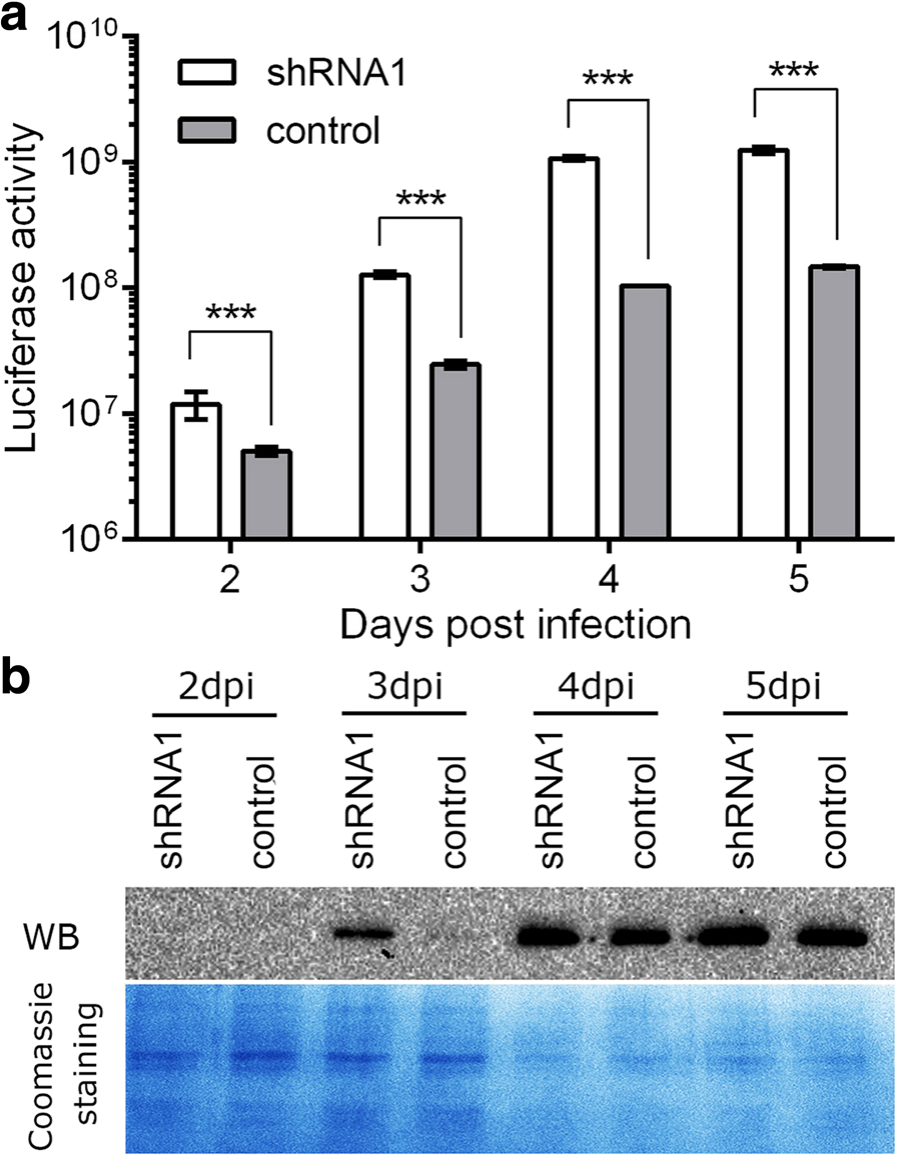
Expression of firefly luciferase using bacmid carrying the Sf-caspase-1 shRNA1 expression cassette. **a**. Luciferase activity. ***p < 0.001 vs. the control. **b**. Luciferase protein levels detected by Western blot (upper panel). Protein was probed by anti-His monoclonal antibody. The loaded total proteins on the PVDF membrane were visualized by coomassie brilliant blue R250 staining (lower panel)

To investigate different kinds of recombinant protein production in the Sf-caspase-1 repressed BEVS, firefly luciferase, an oxidative enzyme that can produce bioluminescence, was expressed as a reporter. This enzyme requires the participation of chaperone components to achieve its correct folding to generate an active enzyme [40]. By Western blot, the protein levels of luciferase in the apoptosis suppressed cells were obviously higher than the control at 2 and 3 dpi but similar as the control at 4 and 5 dpi, in agreement with the expression of fluorescent proteins GFP and DsRed (Fig. 4b). When the enzyme activity was examined, the luciferase level was several-fold higher in the Sf-caspase-1 suppressed cells than the control group at 2 and 3 dpi. At 4 and 5 dpi, the difference of luciferase activity between the two groups reached about ten-fold (Fig. 4a), even though no obvious difference between them was detected by Western blot, suggesting that apoptosis suppression resulted in improved protein folding rather than translation efficiency at the late stage of baculovirus infection.

## Discussion

Previous studies demonstrated enhanced protein production in caspase-1 suppressed cell lines even after 4 dpi [30, 31, 33]. Instead of making stable cell lines expressing siRNA to interfere with caspase-1, we delivered shRNA using a baculovirus vector, so cell apoptosis would only be suppressed during a baculovirus infection but not beforehand. GFP and different enzyme reporters were used in the previous studies, however, only fluorescence intensity and enzyme activities were reported. It would be interesting to see if the two different gene delivery approaches or the detection methods contributed to the discrepancy.

BEVS has been used in many labs as a routine implementation for recombinant protein production. In structural and biophysics studies, it has become the leading platform in producing diffraction-quality proteins among eukaryotic expression systems in recent years [41]. The baculovirus vector made in this study could suppress cell apoptosis and improve protein folding and the production of active proteins, therefore it could be a useful tool to satisfy the increasing demand for active proteins in structural research and in the pharmaceutical industry. The vector may also be adapted for the improvement of protein expression in different insect cell lines that can be infected by AcMNPV. The work is undergoing to engineer the AcMNPV bacmid to target the conserved regions of Sf-caspase-1 and Tn-caspase-1, so that the vector could be used to improve protein production in both Sf and Tn cells.

## Conclusion

In the present study, we generated an AcMNPV shuttle vector carrying shRNA expression cassettes to silence Sf-caspase-1 expression. Infection with baculoviruses made from this vector suppressed the apoptosis of infected *Sf*9 cells, and improved the activity of the expressed exogenous proteins in comparison with the BEVS not expressing shRNA. This baculovirus vector would be useful for the production of various recombinant proteins in insect cells, especially for the expression of active proteins for functional and structural studies or therapeutic applications.

## Abbreviations

AcMNPV: *Autographa californica* multiple nucleopolyhedrovirus
BEVS: Baculovirus expression vector system
Bm: *B. mori*
BmNPV: *Bombyx mori* nucleopolyhedrovirus
BV: budded virus
*chiA*: chitinase gene
dpi: days post-infection
DsRed: *Discosoma sp.* Red
dsRNA: double stranded RNA
GFP: Green fluorescent protein
ODV: occlusion derived virus
PI: Propidium iodide
*polh*: polyhedrin gene
SeMNPV: *Spodoptera exigua* multiple nucleopolyhedrovirus
Sf: *S. frugiperda*
Sf-caspase-1: *Spodoptera frugiperda* caspase-1
shRNA: short-hairpin RNA
TCID_50_: 50% tissue culture infective dose
Tn: *T. ni*
*v-cath*: v-cathepsin gene
